# Certain Adenylated Non-Coding RNAs, Including 5′ Leader Sequences of Primary MicroRNA Transcripts, Accumulate in Mouse Cells following Depletion of the RNA Helicase MTR4

**DOI:** 10.1371/journal.pone.0099430

**Published:** 2014-06-13

**Authors:** Jane E. Dorweiler, Ting Ni, Jun Zhu, Stephen H. Munroe, James T. Anderson

**Affiliations:** 1 Department of Biological Sciences, Marquette University, Milwaukee, Wisconsin, United States of America; 2 DNA Sequencing and Genomics Core, Genetics and Development Biology Center, National Institutes of Health, National Heart Lung and Blood Institute, Bethesda, Maryland, United States of America; University of Florida, United States of America

## Abstract

RNA surveillance plays an important role in posttranscriptional regulation. Seminal work in this field has largely focused on yeast as a model system, whereas exploration of RNA surveillance in mammals is only recently begun. The increased transcriptional complexity of mammalian systems provides a wider array of targets for RNA surveillance, and, while many questions remain unanswered, emerging data suggest the nuclear RNA surveillance machinery exhibits increased complexity as well. We have used a small interfering RNA in mouse N2A cells to target the homolog of a yeast protein that functions in RNA surveillance (Mtr4p). We used high-throughput sequencing of polyadenylated RNAs (PA-seq) to quantify the effects of the *mMtr4* knockdown (KD) on RNA surveillance. We demonstrate that overall abundance of polyadenylated protein coding mRNAs is not affected, but several targets of RNA surveillance predicted from work in yeast accumulate as adenylated RNAs in the *mMtr4*KD. microRNAs are an added layer of transcriptional complexity not found in yeast. After Drosha cleavage separates the pre-miRNA from the microRNA's primary transcript, the byproducts of that transcript are generally thought to be degraded. We have identified the 5′ leading segments of pri-miRNAs as novel targets of *mMtr4* dependent RNA surveillance.

## Introduction

Transcriptional activity in mammalian genomes, once considered limited, is now being viewed differently as more sophisticated techniques are developed and used to interrogate transcriptomes. The sheer abundance of regulatory RNAs (rRNA, snRNA, snoRNA and tRNA) fostered their early discovery and characterization [1]–[6], whereas high-throughput sequencing (HTS) methods continue to uncover an ever increasing population of rare RNAs. The use of HTS to survey mRNA reveals extensive transcript diversity due to alternative splicing, as well as use of alternative transcription start and polyadenylation sites [7]–[9]. These methods have also demonstrated the vast extent to which sequences previously characterized as ‘junk DNA’ are transcribed, accelerating the discovery, classification and characterization of a wide array of non-coding RNAs (ncRNAs) [10]–[19].

Following transcription, RNAs are routinely subject to a series of processing steps ranging from internal cleavage, trimming of ends, RNA editing, and in some cases, the introduction of covalent modifications into the nascent transcript. The production or processing of an RNA occasionally results in the introduction of errors, whereby important features critical to RNA function are disrupted. These defective RNAs can interfere with normal cellular functions, and have been linked to a host of diseases, most prominently neurodegenerative diseases and cancer [20]–[25].

To prevent accumulation or deployment of defective RNAs, eukaryotes invoke RNA surveillance as a “quality control” step that can identify and destroy aberrant RNAs [26]–[30]. Surveillance also recycles processed RNA intermediates for use in de novo rounds of transcription. Once mRNA leaves the nucleus, additional quality control occurs in the cytoplasm (Reviewed in [31]).

Extensive work in yeast led to the discovery of two multi-subunit complexes that are central to nuclear RNA surveillance: the nuclear exosome [32], [33], and TRAMP (Trf4/Air2/Mtr4 Polyadenylation) [29], [34], [35], which identifies and targets RNAs for degradation via the exosome. The exosome contains two ribonucleolytic proteins (Rrp6p and Rrp44p) together with a core of nine structural proteins. Both ribonucleolytic proteins have 3′→5′ exonuclease activity, but Rrp44p also contributes endonuclease activity [36], [37]. The TRAMP complex consists of three subunits: a non-canonical poly(A) polymerase (Trf4p, or Trf5p) that marks byproduct or defective RNAs by appending a poly(A) tail [38]–[40], a Zn-knuckle RNA-binding protein (Air2p, or Air1p; [41]) and an ATP-dependent RNA helicase (Mtr4p, which is also known as Dob1p, or Skiv2l2p) capable of unwinding target RNAs to facilitate degradation by the exosome [34]. These complexes work in concert to eliminate RNAs ranging from hypomodified tRNA [26], byproducts of rRNA processing such as the 5′ external transcribed spacer (5′ ETS; [33]) and even cryptic unstable transcripts (CUTs; [42], [43]).

The surveillance and degradation machinery appears to be well conserved. Exosome complexes are found from archaea to humans [44]–[46], and homologs of the proteins that comprise the TRAMP complex are widely conserved among eukaryotes [47]–[52]. Experimental exploration of RNA surveillance has expanded to mammalian systems relatively recently [50], [51]. One study in humans suggests a strong division of labor in targeting various RNAs to the exosome. Localization analyses suggest that the TRAMP complex may be restricted to the nucleolus, whereas the Nuclear Exosome Targeting (NEXT) complex is excluded from the nucleolus [49]. The common component in both of these complexes is the hMTR4 protein, which exhibits a strong interaction with the hRRP6 protein of the exosome [49]. While RNA targets of TRAMP and the exosome have been extensively characterized in yeast, similar knowledge is relatively scarce in mammalian systems. The transcriptome in mammals is more complex than yeast, both within protein coding genes as well as intergenic regions, now more fully appreciated as transcriptionally active regions producing numerous ncRNAs of mostly unknown function [10], [15], [19]. Thus, the identification and characterization of poly(A)+ RNAs that accumulate upon depletion of a TRAMP homolog subunit, or protein(s) required for RNA surveillance, is useful as an initial exploration of post transcriptional control of RNA expression in mammals.

To identify RNA targets of *Mus musculus* nuclear RNA surveillance, we used a small interfering RNA (siRNA) to deplete the RNA dependent ATPase component of the TRAMP and NEXT complexes, designated as *Skiv2l2* (*mMtr4*) in mouse N2A cells grown in culture. We hypothesized that targets of mammalian TRAMP or NEXT complexes would accumulate in the *mMtr4*-knockdown (*mMtr4*KD) as depletion of mMTR4 would impede degradation of these targets by the exosome. However, such targets should be successfully adenylated by the *mTrf4* homolog, PAPD5, such that they could be identified based upon their poly(A) tails.

We used an RNA-seq strategy specifically designed to capture adenylated transcripts and precisely map the 3′ end of the genomic template (PA-Seq; [53]). Our analyses identified poly(A)+ RNAs that accumulate significantly more in the *mMtr4*KD than in the *mControl*KD. Our data support conserved roles for mMtr4 in processing rRNA 5′ETS for degradation, and in snoRNA surveillance. We identify a novel role for mMtr4 in targeting the 5′ leader sequences of microRNAs for degradation.

## Materials and Methods

### Tissue culture and siRNA knockdown

N2A cells (ATCC CCL-131; [54], [55]) were cultured in DMEM with 10% fetal bovine serum at 37C. Freshly passaged N2A cells were plated in 5 ml of serum containing medium at 5×10^5^ cells per 60 mm tissue culture dish and incubated 24 hours prior to transfection with siRNA. Independent culture dishes were transfected in triplicate with a 1 ml suspension of siRNA in Opti MEM medium and Lipofectamine 2000 (Invitrogen) according the manufacturer's recommendations to a final concentration of 67 nM. Pre-designed siRNAs were obtained from Ambion: *skiv2l2* (Cat#177475) targeted to mouse Mtr4, and “Negative control #1” (Cat #AM4611). After 48 hr incubation with the siRNA, medium was removed, and cells from individual plates were harvested. For western blot analyses, cell pellets were resuspended in lysis buffer and quantified using a Bradford assay. For RNA experiments, cells were directly resuspended with TRIzol Reagent, total RNA was isolated according to manufacturer's recommendations, and quantified using a Nanodrop ND-1000 instrument.

### Determination of knockdown efficiency

Efficiency of the siRNA knockdown was confirmed at both RNA and protein levels. Reverse transcription reactions were completed using SuperScript II (Invitrogen) according to manufacturer's recommendations with an oligo-dT_18_ primer. Primer sets were designed to span an intron to minimize the risk that genomic DNA contamination could result in PCR amplification. Moreover, all primer sets were subjected to melt-curve analysis to confirm single amplicon yield, and tested on Mock-RT reactions (no Reverse Transcriptase added) generated for each RNA sample to further demonstrate amplicons were specifically derived from an RNA template. **Table S4** reports all primers used in this study, and includes information regarding amplicon size and intron(s) spanned.

Real-Time PCR reactions were performed in triplicate using iQ SYBR green master mix (BioRad) and equivalent aliquots of cDNA, with cycling reactions carried out in a My iQ thermocycler (BioRad). Real-Time PCR was used to quantify the level of *Mtr4* mRNA relative to *CyclophilinB* in the control versus knockdown cell lines, thereby determining the relative extent to which the target mRNA had been knocked down. Upon confirmation of efficient knockdown of the target mRNA, these total RNAs were further purified for deep sequencing using an RNeasy kit (QIAGEN) with on-column DNaseI digestion.

Western blot analyses were performed using three independent siRNA treated cell cultures to demonstrate that *Mtr4* protein levels were successfully knocked down. Anti-Mtr4 (Skiv2l2) was obtained from LSBio and anti-β actin from Thermo Scientific was used as a loading control.

### PA-seq

Deep sequencing of the control vs. Mtr4 knockdown RNAs was performed largely as described [53], [56] with the notable exception that (5 ug of each) RNA were first fragmented with magnesium, and reverse transcribed using a biotinylated oligo dT_(16)_/deoxyU/TTTVN-3′ primer to selectively identify adenylated RNAs. cDNAs were selected using Invitrogen's MyOne Streptavidin Dynabeads, dephosphorylated using APex Heat-Labile Alkaline Phosphatase (Epicentre), and digested with the USER enzyme (NEB) to selectively cut the RT primer at the Uracil, which released the dephosphorylated cDNAs from the Dynabeads, leaving a three T tag immediately adjacent to the complementary nucleotide representing the 3′ terminal residue of the adenylated RNA. Following this step, and between each of the subsequent modification steps, the released cDNAs were purified using the DNA clean and concentrator-5 kit (ZYMO Research). Modifications included removal/blunt ending of the 3′ poly(A) overhang remaining after USER digestion using T4 DNA polymerase (NEB), A-tailing using an exo- Klenow fragment (Epicentre), and ligation of the Barcoded Y-linker (Illumina) using T4 DNA ligase (NEB). Resulting cDNAs were separated on an agarose gel (E-gel; Invitrogen), and products in the 250–450 bp size range were excised and purified using a gel purification kit (ZYMO Research). Products were amplified for 16 cycles of PCR (initial denaturation of 30 s at 98°C; then 16×10 s at 98°C, 10 s at 67°C, and 30 s at 72°C; and a final extension of 10 min at 72°C) using Phusion DNA polymerase (Finnzymes), and HPLC purified primers from IDT (PE_PCR_AF: 5′- AATGATACGGCGACCACCGAGATCTACACTCTTTCCCTACACGACGCTCTTCCGATCT-3′; PE_PCR_BR: 5′- CAAGCAGAAGACGGCATACGAGATCGGTCTCGGCATTCCTGCTGAACCGCTCTTCCGATCT-3′). Products for sequencing were size selected (∼250–400 bp) from an agarose gel.

Paired-end sequencing was completed on an Illumina HiSeq2000 instrument. Raw sequencing reads from the Illumina platform were sorted based upon perfect match to a sample specific barcode (at the first 5 bases of read 1), trimmed, and finally split into separate fastq files for the 3′ and 5′ paired-end reads. Project data have been deposited to the NIH short read archive (SRP041394).

### Mapping of Paired-End Reads

Illumina sequencing data was sorted relative to barcode, and trimmed accordingly. The 3′ end of the sequence was evident based upon the presence of the paired end read that initiated with three T's. Paired end mapping to the mouse genome (mm9) was completed largely as described [56] – briefly: using the Burrows-Wheeler Aligner (BWA) mapping algorithm [57] to identify positions within the genome that differ by no more than 2 bp for the 3′ read, include a corresponding match to the 5′ read in the correct orientation (k = 2 & n = 2). Each paired end read that was successfully mapped in this manner was reported as a ‘hit’ for adenylation being initiated at the specific nucleotide in the genome that corresponded with the nucleotide adjacent to the initiating TTTs of the 3′ read. Sequence reads that corresponded with highly repetitive genomic elements were ignored. Paired end reads that did not map to a unique genomic position, but matched fewer than ten genomic positions, were each randomly assigned to one of those genomic positions. In general, adenylation sites with fewer than five mapped sequencing reads were ignored.

### Identification of Adenlyation Peaks

Clusters (a.k.a. peaks of adenylation) were identified largely as previously described [56] using the F-Seq program [58] with the exception that Narrow Peaks were defined as those whose 95% interval was less than 10 bp wide. PA clusters that correspond to a string of adenosines (either six continuous, or 15 out of 20 nt) encoded in the genomic DNA were removed from future analysis. Although their identification within our data suggests that these genomic regions are transcribed, they cannot be definitively attributed to 3′ adenylation versus template adenosines potentially internal to the relevant transcript. A series of Python scripts and the Galaxy portal (main.g2.bx.psu.edu) were used for additional comparative analyses among the sequencing replicates [59]–[61].

### Data Normalization

All data were normalized to account for the sometimes significant difference in sequencing depth between sequencing runs for each of the knockdowns. The total number of PA ‘hits’ at a given genomic location were normalized to reads per million for that knockdown sequencing run. Rather than pooling the sequencing results from biological replicates, most data are reported from one sequencing run (samples designated -1 in **Table 1**) where the total number of reads were comparable among the knockdowns, but the additional sequencing run always exhibited consistent trends.

**Table 1:** Summary of paired-end sequencing experiments.

siRNA knockdown	Raw reads	Mapped reads	Percent mapped reads	Total genomic positions
*mMtr4–1*	15,135,078	10,853,534	71.7%	651,551
*mControl–1*	16,348,780	11,708,310	71.6%	652,128
*mMtr4–2*	40,464,271	34,308,545	84.8%	1,124,968
*mControl–2*	9,124,467	7,195,942	78.9%	582,256

### Molecular validation of differential polyadenylated transcripts

Abundance of adenylated transcripts in the *mMtr4*KD vs. *mControl*KD was independently validated using qPCR. RNA was reverse transcribed using oligo-dT_18_ and either M_MLV (Promega) or SuperScript II (Invitrogen) reverse transcriptase according to manufacturer's recommendations. Equivalent aliquots of each RT reaction were used in triplicate qPCR reactions assaying the levels of adenylated U3 snRNAs, and of the 5′ leader vs. the pri-miRNAs of several miRNAs. In addition to melt-curve analysis to confirm single amplicon yield, real-time amplification data were also analyzed using the DART (Data analysis for Real-Time) platform [62], which uses the kinetics of the real-time reaction to determine the efficiency and consistency of amplification for each primer set. After validating each set of primers for reasonable consistency and efficiency, Ct values determined by the MyIQ software were used to calculate the relative ΔΔCT statistics for each amplicon relative to *CycB* as a housekeeping control gene. Oligonucleoltide primers used for PA-Seq data validation are also included in **Table S4**.

### TaqMan assay of mature miRNA abundance

Abundance of the mature mir322 miRNA was quantified relative to U6 snRNA using their respective TaqMan assays (Applied Biosystems) performed according to manufacturer's recommendations.

## Results

### siRNA Knockdown of *mMtr4* in mouse N2A cells

**Figure 1:**
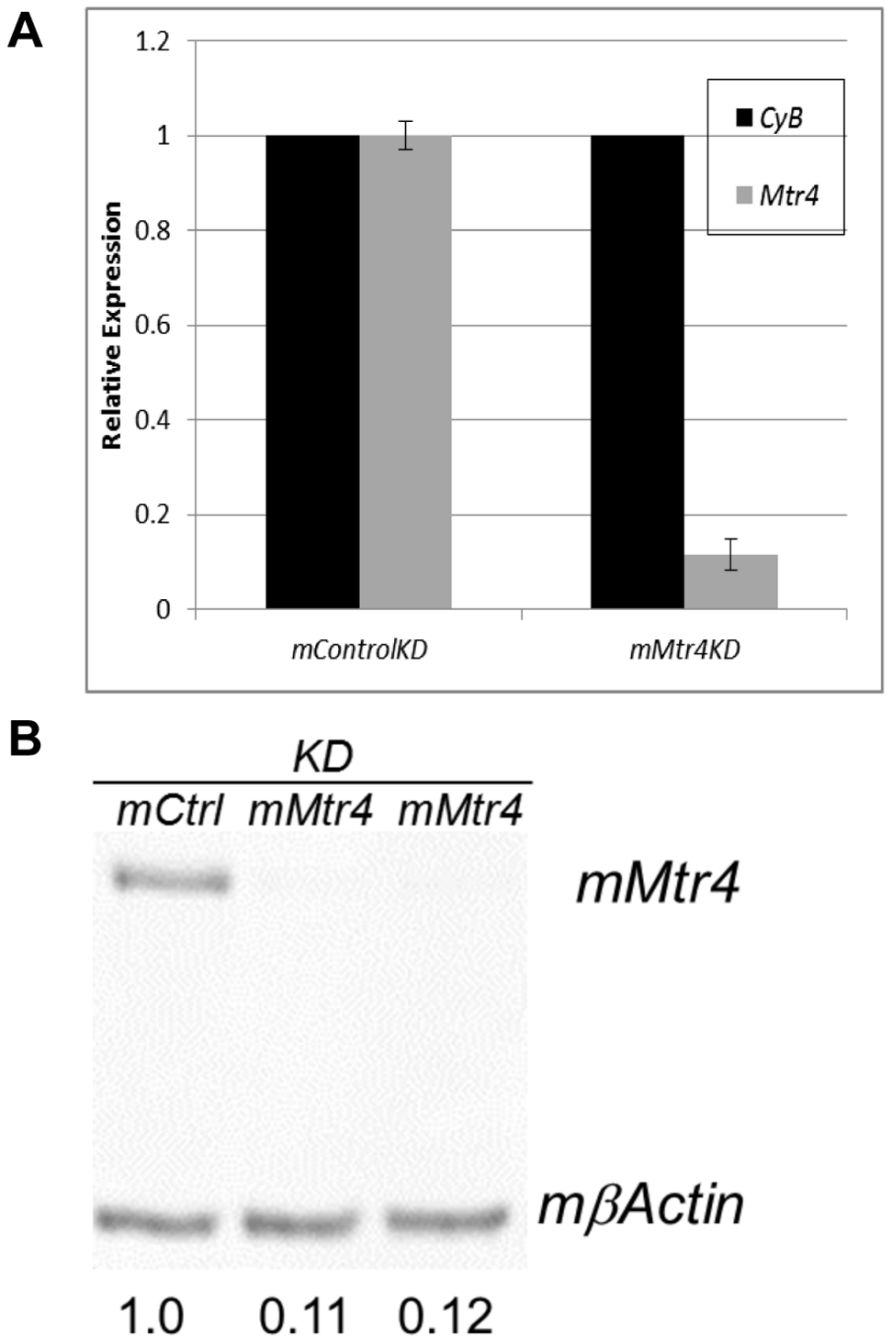
Efficent siRNA knockdown of *mMtr4* mRNA and protein. **A**) Expression of *mMtr4* relative to *mCyB* as a housekeeping control gene in cells treated with Control and mMtr4 siRNAs, respectively. Relative expression was calculated using the ΔΔCt method. Error bars represent standard deviation of three KD experiments. **B**) Western blot of total protein from siRNA treated N2A cells showing level of mMtr4 protein relative to βActin. Comparable numbers were obtained for three independent knockdowns.

N2A cells transfected with siRNA specific to *mMtr4* were assayed relative to cells transfected with a control siRNA. RNAs targeted for exosome degradation by fully functional TRAMP and NEXT complexes should be rare and largely undetectable in control knockdown cells, whereas these same RNAs should accumulate in cells depleted for *mMtr4*. In contrast, we hypothesized that canonical polyadenylation of messenger RNA would be unaffected by the *mMtr4*KD. Thus, adenylated RNAs that are substantially more abundant in the *mMtr4*KD are presumed to be substrates of mMTR4, and possible targets of the mammalian NEXT or TRAMP complexes. The ideal concentration and corresponding knockdown efficiency for individual siRNAs were empirically determined using qRT-PCR assays of the target mRNA (**Fig. 1A**). Immunoblots confirmed successful knockdown of the *mMtr4* protein (**Fig. 1B**).

RNA samples from two replicate knockdowns were used for high-throughput paired-end sequencing designed to specifically capture the 3′ ends of adenylated RNAs. The paired-end reads were mapped to the mouse genome assembly using BWA (See Materials and Methods). Roughly 75% of raw sequence reads were successfully mapped to unique sites in the genome (**Table 1**). Comparison of the two *mMtr4*KD replicates, sequenced to different depths, demonstrates that these sequencing data approach saturation. The ratio of mapped reads between *mMtr4-1* and *mMtr4-2* is 3.15, while the ratio of total genomic positions mapped between the same samples is only 1.73. All data were normalized to account for differences in sequencing depth. As described in the next section, comparisons between replicate KDs demonstrated reproducibility of the PA-seq data. Within each replicate sequencing experiment, the *mMtr4*KD and *mControl*KD, were compared to identify similarities and differences in the respective populations of adenylated RNAs. The replicate sequencing experiments exhibit a strong positive correlation for all target RNAs reported, but unless indicated otherwise, all data shown are from one dataset (those samples designated by −1).

### Polyadenylation of messenger RNAs are largely unaffected by depletion of *mMTR4*

**Figure 2:**
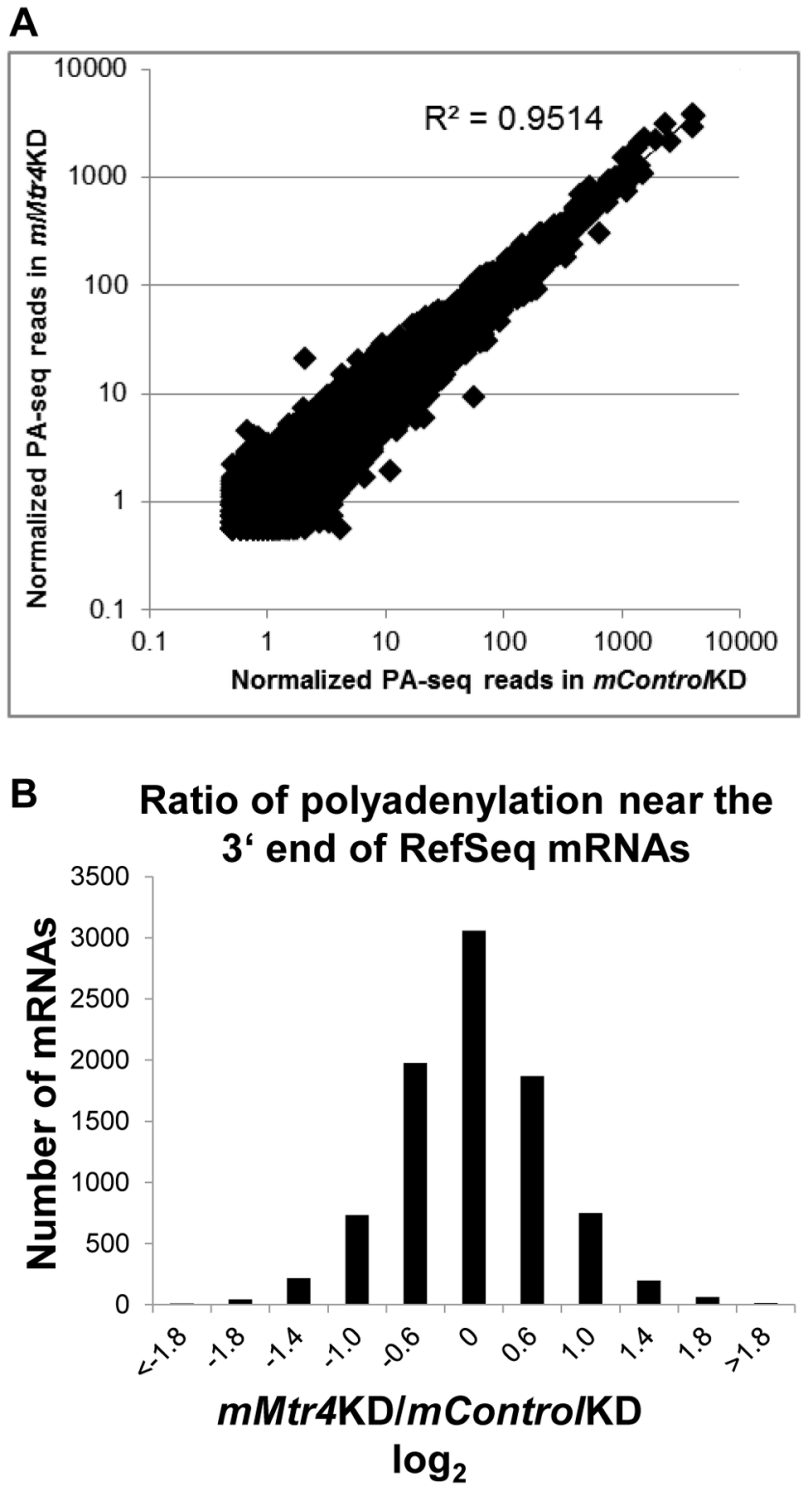
Knockdown of *mMtr4* does not significantly alter polyadenylation of protein coding mRNAs. **2A**). Dot Plot of PA-seq data from the *mMtr4*KD versus *mControl*KD for 8,935 protein coding RefSeq genes. **2B**). Histogram reporting log_2_ transformed ratios of *mMtr4*KD/control reads. A value of 0 equals no difference, +1 equals a two-fold difference, +2 equals a four-fold difference, and positive values indicate that polyadenylated transcripts are more abundant in *mMtr4*KD, whereas negative values indicate that they are more abundant in the *mControl*KD. The bin labeled 0 includes log_2_ ratios ranging from +0.2; all other bins include values expanding by +0.4 in either direction, and labeled according to the outer boundary of that bin relative to zero.

To test the hypothesis that *mMtr4*KD should not affect the abundance of protein coding mRNAs, normalized polyadenylated sequencing reads terminating within +50 bases of the annotated 3′ end of RefSeq protein coding genes were identified for each KD. For each of the KDs, roughly 8500 RefSeq genes are represented in the PA-seq data with a minimum of five sequencing reads at the modal position. Data from the modal position were used to test the experimental reproducibility between sequencing experiments. A dot plot of the two *mControl*KDs (*mControl-1* vs. *mControl*-2), with RNA isolation completed in parallel but library construction completed independently, demonstrated reproducibility of the PA-Seq data (R^2^ = 0.87). The *mMtr4*KD and *mControl*KD sequencing reads of 8,935 RefSeq modal PA-sites are shown as a dot plot (**Fig. 2A**), and the ratio of *mMtr4*KD versus *mControl*KD reads are presented as a histogram (**Fig. 2B**). The dot plot of *mMtr4*KD vs *mControl*KD indicated little variability in polyadenylation of the RefSeq protein coding mRNAs under conditions where *mMtr4* has been depleted (**Fig. 2A**, R ^2^ = 0.95). The histogram provides an alternative view of the data for *mMtr4*KD vs *mControl*KD, revealing a normal distribution, with ∼94% of the RefSeq genes used in the analysis exhibiting less than a two-fold difference (|log_2_|<1) between *mMtr4*KD and *mControl*KD. The ∼6% of RefSeq genes falling outside the two-fold difference are largely observed at the lowest expression levels (**Fig. 2A**), consistent with higher variability at lower detection thresholds. Moreover, when examined in the replicate PA-Seq data set, the relative expression of these same RefSeqs in the *mMtr4*KD and *mControl*KD were equally split among three categories: equivalent expression levels (<2-fold difference), comparable difference (>2-fold difference), or an inverse correlation (>2-fold difference in the opposite direction). The data were also examined for evidence of differential polyadenylation position usage between the *mMtr4*KD and *mControl*KD. Although RNAs corresponding to alternative positions exist within the data, no significant differences in the relative distribution of site usage were apparent.

### The 5′ ETS of rRNA is a conserved target of yeast and mammalian RNA surveillance

**Figure 3:**
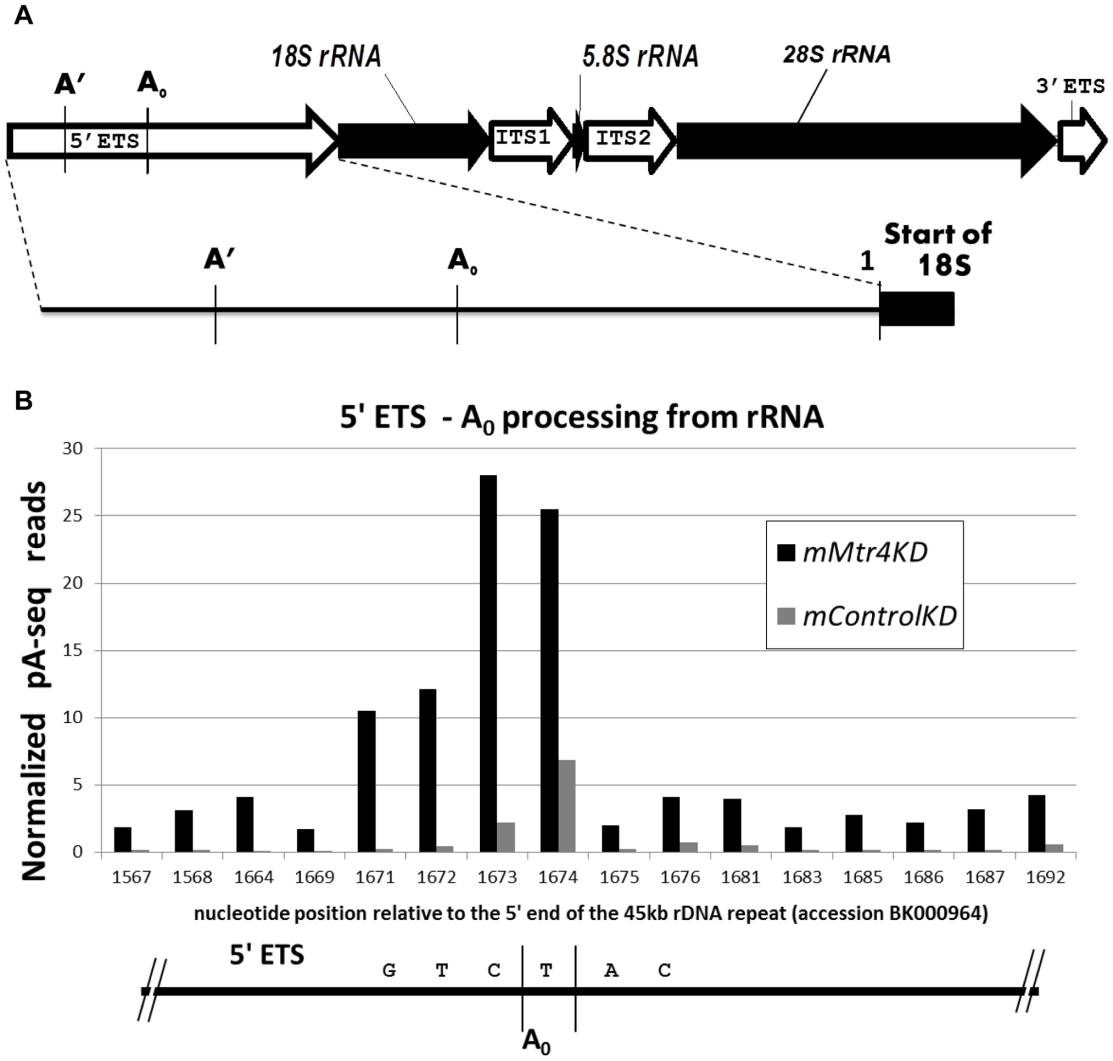
Adenylated 5′ETS transcripts accumulate in *mMtr4*KD. **3A**) Schematic of precursor rRNA and relevant processing sites. The above schematic depicts the External and Internal Transcribed Spacers (ETS and ITS, respectively) of mouse pre-rRNAs. The 18S, 5.8S, and 28S rRNAs are shown as filled black arrows, whereas the External and Internal Transcribed Spacers (ETS and ITS, respectively) are shown as unfilled arrows. The 5′ETS is enlarged below to show the approximate locations of processing sites A′, A_0_, and the 18S junction (site #1). Processing site nomenclature according to Kent et al 2009. **3B**). Histogram reporting site specific adenylation of 5′ETS transcripts in the *mMtr4*KD relative to *mControl*KD. Relative adenylation, normalized as reads per million, is reported for specific nucleotide positions within the 5′ ETS. The sites exhibiting the highest levels of adenylation correspond with the two adjacent nucleotide positions identified as corresponding to A_0_ rRNA processing sites by Kent et al 2009. Nucleotide positions are reported as the distance from the 5′ end of the pre-rRNA (BK000964).

Convinced that changes in amplitude of PA-seq reads between *mMtr4*KD and *mControl*KD at a given locus would be the result of differences in RNA abundance, we focused on one of the known targets of the yeast TRAMP complex; the 5′ ETS of pre-rRNA. The 5′ ETS has also been observed to accumulate in human tissue culture cells depleted of *hMtr4* [49]. The PA-seq data demonstrate that the mouse 5′ ETS accumulates in the *mMtr4*KD relative to *mControl*KD. Processing intermediates of the mouse pre-rRNA 5′ ETS include the region from the transcription start to the A′ processing site, from A′ to A_0_, and from A_0_ to the 18S cleavage junction ([63], [64]; **Fig. 3A**). Accumulation of A_0_ terminating fragments (+2 bp) is 8-fold higher in the *mMtr4*KD than the *mControl*KD (**Fig. 3B**). In contrast, adenylated fragments terminating at A′ or the 18S junction were below the PA-seq detection threshold in any of our PA-seq libraries (**Fig. S1**; also see discussion). Thus, accumulation of 5′ ETS adenylated RNAs in the *mMtr4*KD is specific to the A_0_ processing site. We conclude from this observation that degradation of the 5′ ETS at the A_0_ site is affected by depletion of *mMtr4*.

### U3 snoRNA adenylated transcripts accumulate in cells depleted of *mMtr4*

**Figure 4:**
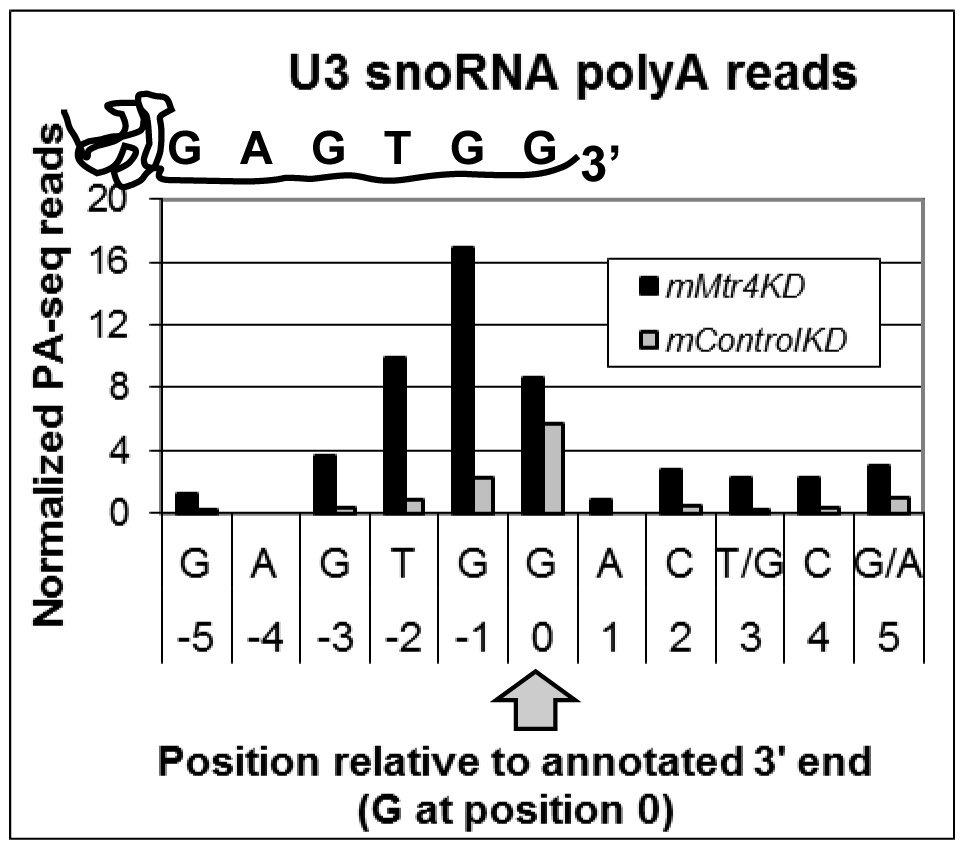
Adenylated U3 snoRNAs from Chromsome 11 (U3B.1-U3B.4) differentially accumulate in the *mMtr4*KD. This histogram reports the level of adenylation at individual positions of U3B snoRNAs. Positions are shown relative to the 3′ end of the snoRNA, as indicated schematically above, and by an arrow below the x-axis. Adenylated RNAs terminating immediately 5′ (positions −1 thru −3) relative to the canonical 3′ end of the snoRNA (position 0) are cumulatively 9-fold more abundant in the *mMtr4*KD relative to the *mControl*KD.

Many of the snoRNAs function as guides directing accurate processing or modification of precursor rRNA transcripts to release 18S, 5.8S and 28S rRNAs, and facilitate their maturation and function through nucleotide modification [65]–[68]. Specifically, U3 snoRNPs form duplexes at several sites along the 5′ ETS to direct endonucleolytic cleavage [64]. There are four identical copies of the U3B snoDNA (U3B.1-U3B.4) gene located in tandem within the 2^nd^ intron of Tex14 mRNA (NM_031386) on mouse chromosome 11 [69]–[71]. We observed accumulation of adenylated U3 snoRNA transcripts within our PA-seq data corresponding to all four copies of U3B, and sought to characterize these data further. First, we determined that all of the 5′ paired-end reads map within the 214 bp of the mature snoRNA. This suggests that the reads derive from *bona fide* U3B processing rather than read through transcription. We report the mapped PA-seq data pooled from all four U3B loci (**Fig. 4**). The level of U3 snoRNAs adenylated precisely at their annotated 3′ ends was nearly equivalent in *mMtr4*KD vs. *mControl*KD (**Fig. 4**). These data may indicate that routine turnover of aberrant or perhaps even mature copies of U3 snoRNA occurs via an adenylation dependent pathway. In stark contrast, shorter U3 snoRNAs adenylated at nucleotide positions immediately 5′ of the mature snoRNA 3′ end are 9-fold more abundant in the *mMtr4*KD relative to the *mControl*KD (**Fig. 4** **, Fig. S2**). Slightly longer U3 snoRNAs are also observed, and are generally more abundant in the *mMtr4*KD. A comparable distribution of adenylated transcripts from the U3A locus on chromosome 10 are observed (data not shown). Given that normal U3 processing involves 3′→5′ exosome-mediated trimming [72], these data may indicate that U3 snoRNAs are occasionally ‘over-trimmed’, and that such errors are eliminated by MTR4-facilitated degradation.

Quantitative RT-PCR was used to confirm differential accumulation of adenylated U3 snoRNA in *mMtr4*KD relative to *mControl*KD. Total RNA from two replicate knockdowns was reverse transcribed using oligo-dT, and subjected to qPCR to measure the level of poly(A)+U3 snoRNA in the *mMtr4*KD and *mControl*KD using U3 specific primers. Given that oligo-dT primed reverse transcription (RT) would not distinguish between full-length and shortened adenylated U3 snoRNAs, our qPCR results reflect the sum of all adenylation sites reported in **Fig. 4**. Thus, the observed ∼5-fold difference between *mMtr4*KD and *mControl*KD by qPCR is consistent with the PA-seq data (**Table S1**).

### Adenylated transcripts accumulate in the *mMtr4*KD

To identify additional targets of mMTR4 mediated degradation, the PA-seq data was analyzed using the F-Seq feature density estimator [58] to identify clusters, or peaks, of adenylation. Peak data were filtered to eliminate sequencing reads corresponding to templated adenosines (see Methods). All remaining peaks were categorized as corresponding to annotated mRNA polyadenylation sites, repetitive genomic elements (RepeatMask track), or neither. Peaks in the neither category were compared to the *mControl*KD to identify RNAs that exhibit differential accumulation. Those exhibiting the largest ratios are shown in Table 2, along with information regarding their relative genomic context. Strikingly, four of the top five RNAs exhibiting increased accumulation in the *mMtr4*KD map to a microRNA or microRNA host gene.

**Table 2:** Adenylated RNAs that accumulate in *mMtr4*KD.

chrm	strand	mm9 mode coordinate	*mMtr4*KD*	*mCtrl*KD*	Mtr4/Ctrl Ratio	
chr9	**+**	122592015	65	1	65	mir138 5′ leader
chrX	**−**	50407504	250	4	62.5	mir322 5′leader
chr9	**+**	40613015	332	43	7.72	last intron of Hspa8**
chr15	**+**	85537754	40	6	6.67	let7b 5′ leader
chrX	**−**	50406290	136	24	5.67	mir322 3′ UTR terminus
chr6	**−**	48474268	37	10	3.70	unknown??***

* Data are reported as total raw reads from replicate −1, and summed over positions +2 bp relative to the peak mode.

** Modal position precisely corresponds to the 3′ end of the Hspa8 intron, which contains the U14 snoRNA terminating 95 bp upstream.

*** Maps within a partial Y-box binding protein (oxyR) mRNA and AS rel to ∼3rd intron of Zfp862.

### Adenylated transcripts associated with the mir322 host gene accumulate in the *mMtr4*KD

**Figure 5:**
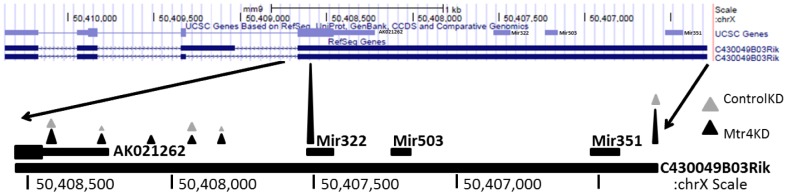
Abundant PA-seq reads map near mir322. The top diagram shows the mir322 - mir503 – mir351 cluster and associated RIKEN transcripts on chromosome X. Note that the chromosomal orientation has been reversed such that the mm9 chromosomal coordinates are in decreasing order from left to right because the transcripts are on the minus strand. The rightmost portion of this region has been expanded below to highlight both the location and relative abundance of adenylation in the *mMtr4*KD (black) vs. *mControl*KD (grey).

The above analyses identified two significant peaks of adenylation on chromosome X corresponding to a full length non-coding cDNA (RIKEN-C43009B03RIK) containing a miRNA cluster (mir322-mir503-mir351). To gain a better appreciation for the distribution of adenylation across this cDNA, we examined all F-Seq peaks across the 4 kb span of C43009B03RIK. Distribution of the *mMtr4*KD peaks reveal three prominent areas of adenylation in this region of chromosome X: 1) at the 3′ annotated end of cDNA C43009B03RIK, 2) a loose clustering near the 3′ annotated end of a shorter RIKEN cDNA AK021262 that encodes a hypothetical 92aa protein but does not include the miRNA cluster, and 3) at mir322 (**Fig. 5**). The presence of PA-seq peaks in our data corresponding to the annotated 3′ ends of both RIKEN cDNAs indicates that our technique is effectively interrogating transcriptional activity at this genomic locus. Consistent with the existence of two RIKEN cDNAs at this locus, our data also indicate there are two outcomes of transcription starting at the 5′ end of this genomic locus; one transcription unit which includes the miRNA322, 503 and 351 cluster and a shorter one that does not include the miRNAs.

**Figure 6:**
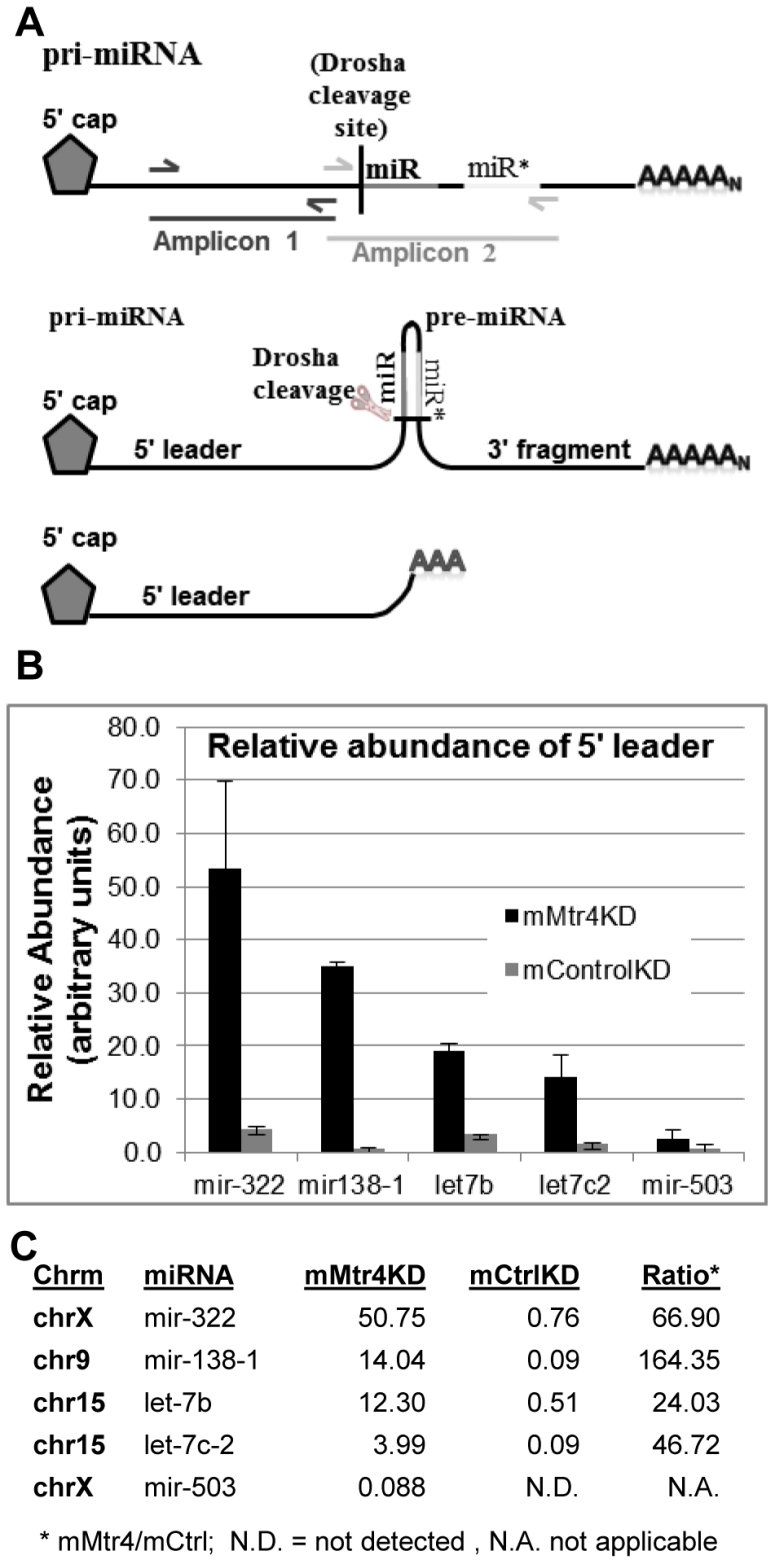
Adenylated 5′ leader sequences from pri-miRNAs accumulate in the *mMtr4*KD. **A**. Schematic depicting relevant aspects of miRNA processing. Primary miRNA transcripts are produced by RNA polII and include both a 5′ cap and poly(A) tail. The miRNA and complementary mir* contribute to formation of a hairpin, and Drosha cleaves near the base of the hairpin to liberate the pre-miRNA [103]. *mMtr4*KD PA-seq identified adenylated 5′ leader transcripts whose 3′ end specifically maps to the Drosha cleavage site. qRT-PCR validation of these data compared amplification results for primers within the 5′leader (eg. Amplicon 1, depicted on uppermost diagram) to primers that span the Drosha cleavage site (Amplicon 2) as a control for pri-miRNA abundance. **B**. Relative expression of miRNA 5′ leader sequences was calculated using the ΔΔCt method from triplicate reaction realtime PCR data. All reactions were performed in triplicate, further averaged over 3-4 independent qPCR runs for each miRNA 5′ leader, and shown relative to parallel qPCR reactions detecting Cyclophilin B mRNA. All Y-axis values represent relative arbitrary units. **C**. Combined PA-seq data for miRNA 5′ leader sequences reported as reads per million, and ratio of *mMtr4*KD over *mControl*KD.

Strikingly, the substantial cluster of adenylated RNAs around the 5′ end of mir322 is notably absent in the *mControl*KD (**Fig. 5**). The mode of this highly active adenylation cluster in the *mMtr4*KD precisely corresponds with the Drosha cleavage site (**Fig. 6A**). Directed interrogation of the *mControl*KD data identified only three PA-seq reads at this position, indicating that there is a 67-fold increase for these adenylated transcripts in the *mMtr4*KD. Drosha cleavage of the primary miRNA transcript would release three distinct products; the pre-miRNA, a 5′ leader fragment (hereafter referred to as the 5′ leader) and a 3′ fragment (**Fig. 6A**). In the case of mir322, the 3′ fragment will contain two additional microRNAs, mir503 and mir351, which may subsequently influence the fate of that fragment (discussed below). The notable PA-RNAs that accumulate in the *mMtr4*KD (**Table 3**), represent the 5′leader, suggesting that mMTR4 may play a role in targeting the 5′leaders of microRNAs to the exosome for degradation, in an adenylation dependent manner.

**Table 3:** Combined PA-seq data corresponding to miRNA 5′ leaders (with ≥25 reads)*.

chromosome	microRNA	*mMtr4*KD	*mControl*KD**
chrX	mmu-mir-322	1257	7
chr15	mmu-let-7b	422	6
chr9	mmu-mir-138-1	377	1
chr15	mmu-let-7c-2	99	1
chr5	mmu-mir-106b	58	3
chr1	mmu-mir-26b	46	1
chr6	mmu-mir-29a	37	N.D.
chr14	mmu-mir-124-1	35	N.D.
chrX	mmu-mir-463	31	N.D.
chr6	mmu-mir-29b-1	28	N.D.

* Data are reported as total raw reads combined from both biological replicates, and summed over positions +2 bp relative to the predicted Drosha cleavage site.

^**^Raw reads from *mControl*KD are not directly comparable to *mMtr4*KD due to ∼3-fold higher coverage in one of the sequencing experiments. Figure 6C provides normalized values for the top four miRNA 5′ leaders. N.D.  =  not detected.

The vast increase in abundance of adenylated mir322 5′ leader sequences in *mMtr4*KD relative to the *mControl*KD, along with a modest 6-fold increase in transcripts adenylated at the annotated 3′ end of the full length RIKEN cDNA, might reflect increased expression of mir322. To determine whether the *mMtr4*KD causes differential expression or accumulation of mir322, we employed a TaqMan miRNA assay to quantify the relative levels of the mature mir322-5p microRNA in *mMtr4*KD and *mControl*KD RNAs. The mir322 TaqMan assays were normalized to U6 snRNA levels that were assayed in parallel. Analyses in three biological replicates suggest that mature mir322 levels may fluctuate in either direction by as much as two fold in the *mMtr4*KD and *mControl*KD, but that no consistent trend exists (**Table S2**). Although we cannot completely rule out that the mir322-mir503-mir351 cluster is more actively transcribed upon mMTR4 depletion, even a 2-fold increase would seem too small to explain the large, 67-fold increase in adenylated mir322 5′ leader by a mechanism of increased transcription alone. In a related experiment, PA-seq detected a modest increase in the mir322 5′leader and annotated 3′ end of RIKEN-C43009B03RIK cDNA in N2A cells depleted for the exonuclease *mRrp6* (data not shown/unpublished). Taken together, these data specifically support the idea that differences between *mMtr4*KD and *mControl*KD are from impaired degradation of those transcripts, rather than increased transcription.

### MTR4 facilitates degradation of miRNA 5′ leaders

Two additional microRNAs, let7b and mir138-1, display clusters of adenylation for *mMtr4*KD (**Table 2**). Comparable F-Seq analysis using the deeper of the two *mMtr4*KD sequencing data sets (*mMtr4*-2) identified a total of six miRNA peaks, expanding the list to include let7c-2, mir106b and mir26b (**Table 3**). In all cases, the modal position of adenylation maps precisely to the predicted Drosha cleavage site, such that the adenylated RNA corresponds to the 5′ leader. In sharp contrast, no corresponding peaks were identified in the *mControl*KD. In an effort to determine whether the *mControl*KD may contain corresponding 5′ leader sequences below the level of detection by F-Seq, or whether *mMtr4*KD may contain 5′ leader sequences corresponding with additional microRNAs, we asked whether reads in the complete PA-seq data sets map in or around any annotated miRNAs. This analysis identified a small number of PA-seq reads in the *mControl*KD (**Table 3**), and PA-seq reads in *mMtr4*KD corresponding with over 100 additional miRNAs, eighty-five percent of which (105/124) include reads mapping consistent with the predicted 5′ leader terminating at the Drosha cleavage site. Although a majority of the 105 additional miRNAs are represented by fewer than 5 total PA-seq reads, it is difficult to dismiss the correlation with the position of the 5′ leader as coincidental. All miRNA 5′ leader PA-seq data are available in **Table S3**.

Oligo-dT primed qRT-PCR was used to independently test miRNA 5′ leader sequence accumulation in the *mMtr4*KD relative to *mControl*KD. We used the same RNA samples used to generate the PA-seq data, and focused on mir322, mir503, let7b, let7c2 and mir138-1. qRT-PCR analysis of miRNA 5′ leader sequences was analyzed relative to qRT-PCR that spans the junction between the 5′ leader and the pre-miRNA (as depicted by representative amplicons 1 and 2 in **Fig. 6A**). Amplicon 2 serves as a critical control for the level of detectable adenylated pri-miRNA that would be amplified by the primers located within the 5′ leader. qRT-PCR results support accumulation of the miRNA 5′ leader relative to the pri-miRNA for mir322, let7b and mir138-1 (**Figure 6B**). In contrast, qRT-PCR results for amplicon 2 primers spanning the Drosha cleavage site were uniformly low. For comparison, **Figure 6C** presents normalized PA-seq reads for each of these microRNA leaders from both *mMtr4*KD and *mControl*KD, as well as their relative ratio. The qRT-PCR data for let7c2 and mir503 are less dramatic than the other microRNA 5′ leaders, suggesting that the respective pri-miRNAs are more abundant than the 5′ leaders produced by Drosha, either due to less frequent Drosha processing for these miRNAs, or instability of the 5′ leaders of these miRNAs despite knockdown of *mMtr4*.

## Discussion

We have analyzed the effect of depleting *mMtr4* on RNA adenylation in mouse N2A cells using small interfering RNAs (siRNA). We used a PA-seq strategy to selectively amplify and sequence adenylated RNAs. Depletion of *mMtr4* does not alter the abundance of adenylated protein coding mRNAs. In contrast, depletion of *mMtr4* results in the accumulation of a number of adenylated non-coding RNA. Both the rRNA 5′ ETS and U3 snRNA have been previously reported as targets of TRAMP. The accumulation of adenylated 5′ leader transcripts from pri-miRNAs is novel, and suggests a role for *mMtr4* in targeting these transcripts for degradation by the exosome. Together, these results are consistent with observations linking MTR4 homologs to RNA surveillance [47], [49], [73]–[76]. Isolation of these target RNA sequences based upon presence of a poly(A) tail further suggests that, similar to extensive data in yeast [39], [40], [42], [77] and more recent observations in humans [49], at least some mMTR4-mediated RNA surveillance in mouse also involves a Poly(A) polymerase. In contrast, others have observed significant accumulation of adenylated PROMPT RNAs in hMtr4KD human cell lines [49], [78], whereas significant PROMPT accumulation was not detected in our PA-Seq data. This may reflect differences in sensitivity of the respective gene specific RT-PCR vs. PA-Seq methods, or issues related to oligo-dT RT reactions or adenylate tail length.

### The 5′ ETS of pre-rRNA is a conserved target of MTR4-mediated decay in mouse

RNA surveillance has been most extensively characterized in yeast, where the TRAMP complex functions to identify and target specific RNAs for degradation. The yeast TRAMP complex consists of the DExD/DExH box RNA helicase Mtr4p, as well as a poly(A) polymerase (either Trf4p or Trf5p), and a RNA-binding protein containing five Zn-knuckles (either Air1p or Air2p). Although *Mtr4* is an essential gene, repressible or reduced function alleles of *Mtr4* have been used to identify targets of TRAMP and characterize the role(s) played by Mtr4p as part of the TRAMP complex [33], [73], [79]. Allmang and colleagues [33] determined that depletion of *mtr4* resulted in time-course dependent accumulation of the 5′ ETS of yeast rRNA.

We observe significant accumulation of 5′ETS sequences adenylated at the A_0_ processing site in our *mMtr4*KD, consistent with this rRNA byproduct being a conserved target of the mammalian TRAMP complex. Nevertheless, given the relative abundance of rRNA, the frequency with which we observe these transcripts (∼28 reads per million in *mMtr4*KD, **Fig. 3B**) seems surprisingly low. We also explored whether rRNA transcripts 5′ of the A′ processing site, or immediately adjacent to the 18S rRNA (see diagram in **Fig. 3A** **, and Fig. S1**), accumulate in a similar fashion to those at A_0_. Among ∼31 million raw 3′ reads queried, including both the *mMtr4*KD and *mControl*KD, only 3 adenylated transcripts corresponding with the region 5′ of the A′ processing site and no reads corresponding to the 18S proximal segment were observed. Abundant data suggest that pre-rRNA processing should occur frequently at the A′ site [63], [64], [80], although failure to accumulate adenylated fragments terminating at this position in the *mMtr4*KD is consistent with the fact that the analogous portion of the yeast 5′ETS is not degraded by the exosome, but rather by 5′→3′ exonucleases Xrn1p and Rat1p [67], [81]. Overall, substantive accumulation of 5′ETS adenylated RNAs in the *mMtr4*KD is specific to the A_0_ processing site.

### PA-seq reveals a heterogeneous population of adenylated U3 snoRNAs in *mMtr4*KD

snoRNAs have also been observed to be targets of Mtr4p in yeast [33], [82]. Whether Mtr4p is targeting adenylated snoRNAs for degradation, for 3′ end processing by an exonuclease, or some combination of the two has not been explored, but our data would suggest that both may be true in mouse. Detection of U3 transcripts adenylated at or just beyond the annotated 3′ end in the *mControl*KD is consistent with the idea that adenylation is part of the normal U3 life-cycle, from 3′ end maturation to normal turnover of snoRNAs. Derti and colleagues [83] observe adenylated U3A in an assortment of wild-type tissues. The fact that we observe significantly higher levels of adenylated U3 in the *mMtr4*KD, with a substantial increase in transcripts adenylated just 5′ of the annotated 3′ end, is consistent with the concept that mMTR4 functions not only in the normal life-cycle of U3 as observed in normal cells, but also in turnover of defective U3 snoRNAs, such as those that may have been over-trimmed by an exonuclease.

### Nuclear RNA surveillance of miRNA 5′ leaders

It is commonly accepted that extraneous portions of miRNA primary transcripts released during miRNA biogenesis are ‘simply degraded’, but the cellular components responsible for targeting those RNAs for degradation have not been previously identified. Our PA-seq data suggest that mMTR4, possibly as part of the mammalian TRAMP complex, plays a role in directing some of these miRNA byproducts, particularly the 5′ leader, to the nuclear exosome for degradation.

While we were able to detect adenylated 5′ leader sequences corresponding to over 100 miRNAs in the mMtr4 knockdown, only a handful of these accumulate to highly significant levels (**Table 3** **,** **Fig. 6**, and **Table S3**). Interestingly those that accumulate to the highest levels correspond to abundant miRNAs. Both mir322 and let7 are relatively ubiquitous across a wide range of tissues, and among the most abundant microRNAs detected [84]–[86]. Several of the other microRNAs play critical functional roles in neural or brain development, or in cases where their function is as yet unknown, they are abundantly represented in neuronal cells, or during brain development [87]–[95].

Much of the small RNA deep sequencing data in mirbase also suggests that the 5′ most member of putative co-transcribed miRNAs is often more abundant than those progressively 3′ within the cluster [84], [96]–[98], consistent with the idea that Drosha may preferentially liberate specific pre-microRNAs from polycistronic pri-microRNAs, and that in some cases, the remaining transcript may be degraded rather than processed further. Consistent with this observation, miR322 is the 5′ most member of its microRNA cluster.

We explored the genomic context for several more of the most abundant microRNA 5′ leaders (let-7b, let-7c2, mir138-1, mir106b and mir26b) in an effort to determine whether context might influence accumulation of the 5′ leader in the *mMtr4*KD. Indeed, many of the most abundant 5′ leaders correspond to microRNAs presumed to be co-transcribed with other microRNAs. miR138-1 is one exception, residing in an intergenic region of chromosome 9 with no other annotated miRNAs or mRNAs nearby. The other is mir26b, which is found within the fourth intron of a protein coding transcript (NM_153088, Ctdsp1). Similar to mir322, mir106b is the 5′ most microRNA within the mir106b-mir93-mir25 cluster, which is located within intron 13 of the Mcm7 gene. Finally, let-7c2 and let-7b are less than 1.0kb apart and presumed to be part of the same primary transcript. While the 5′ leader sequences for both of these microRNAs accumulate, we observed a greater amplitude of PA-seq accumulating in *mMtr4*KD for the more distal let-7b than for let-7c2, both being appreciably above that seen in the *mControl*KD. Overall, the reason we see preferential (sometimes exclusive) accumulation of the 5′ leader for one member of a miRNA cluster is unknown, but could reflect differences in processing order or efficiency, the possibility that only some of the processing byproducts are adenylated, or RNA structural determinants of the accumulating PA-seq.

Although it remains unclear why specific miRNA 5′ leaders accumulate in the *mMtr4*KD, it is worth noting that we do not observe notable accumulation of a corresponding 3′ fragment that would simultaneously be liberated by Drosha cleavage of the pre-miRNA from the pri-miRNA. One factor that may influence miRNA 5′leader accumulation is the 5′ cap on these RNApolII derived transcripts preventing 5′→3′ exonuclease degradation, whereas the 3′ fragment would be susceptible to both 5′→3′ and 3′→5′ exonuclease degradation. Consistent with this distinction, 5′ proximal versus 3′ proximal RNAs resulting from cotranscriptional cleavage (CoTC) of the human beta-globin gene have alternate fates. The 3′ proximal RNAs are degraded by the Xrn2 5′→3′ exonuclease [99], whereas the 5′ proximal RNAs are subject to exosome mediated degradation [100]. Presence of a 5′ cap could also help explain the correlation that several of the 5′ leaders that accumulate are from the 5′ most members of miRNA clusters thought to be co-transcribed. Overall, no single aspect appears to readily predict which microRNA 5′leaders will accumulate in *mMtr4*KD cells, or the relative extent to which they will accumulate, but a select few are by far the most abundant adenylated RNAs we have thus far identified in the *mMtr4*KD.

### Implications for nuclear RNA surveillance in mammals

Although extensive research has uncovered a wealth of TRAMP substrates in yeast, the absence of microRNAs in yeast and thus our inability to predict this novel finding underscores the importance of expanding research into a wider array of organisms. This finding also reveals the extent to which nuclear RNA surveillance complexes may have evolved to target an ever increasing diversity of substrates as genomes evolve and increase in complexity. Finally, our observation suggests that depleting MTR4 proteins in a wide array of tissues or organisms may be a useful mechanism for characterizing the 5′ ends of pri-miRNAs, such as determining transcription start sites and identifying promoter elements.

Well conserved homologs of Mtr4, Trf4 and Air2 form a TRAMP-like complex in humans, but immunofluorescence localization suggests that the Air2 homolog ZCCHC7 exclusively localizes to the nucleolus [49]. Given that the Air2 protein of yeast is critical for assembly of the yeast TRAMP complex [101], and that ZCCHC7 is the only human protein with significant similarity to the Air2 and Air1 paralogs of yeast, it is unclear how mammalian Trf4 (also known as Papd5) and Mtr4 would collaboratively function outside the nucleolus. Although the NEXT complex exhibits a complementary localization within the mammalian nucleus, and is excluded from the nucleolus, neither Trf4 nor any other poly(A) polymerase co-immunoprecipitated with this complex [49]. Nevertheless, surveillance may involve one or more canonical poly(A) polymerases [78], [102], or the mammalian Trf4 may function autonomously outside the nucleolus [50]. Definitive identification of the poly(A) polymerase(s) responsible for adenylating will further illuminate RNA surveillance in mammalian systems. Overall, our PA-Seq analyses confirm the role of *mMtr4* in adenylation mediated degradation of the 5′ ETS of rRNA and U3 snoRNAs, and suggest that some miRNA 5′ leader sequences utilize *mMtr4* in a similar degradation pathway.

## Supporting Information

Figure S1 **Density of PA-seq reads across the entire 5′ ETS.** Graph depicts the level of adenylation at individual positions across the 4 kb 5′ ETS. Positions for which zero reads were detected in either the *mMtr4*KD or *mControl*KD were omitted for improved resolution of all remaining positions. Positions along the x-axis are not to scale, but several reference points are indicated.
(TIF)

Figure S2 **Density of PA-seq reads encompassing U3B.** Graph depicts the level of adenylation at individual positions across the full length of U3B snoRNA. Data are pooled for the four tandem copies of U3B found on chromosome 11 of the mouse genome.
(TIF)

Table S1 **Quantitative RT-PCR results for U3 snoRNA.**
(XLSX)

Table S2 **Mature mir322 levels exhibit no consistent fluctuations in mMtr4 depleted cells.**
(XLSX)

Table S3 **All miR associated pA-seq reads.**
(XLSX)

Table S4 **All oligos used for qPCR.**
(XLSX)
