# Supplementary figures and images for: Certain Adenylated Non-Coding RNAs, Including 5′ Leader Sequences of Primary MicroRNA Transcripts, Accumulate in Mouse Cells following Depletion of the RNA Helicase MTR4
Source: PLoS One. 2014 Jun 13;9(6):e99430. doi: 10.1371/journal.pone.0099430 (PMC4057207; doi:10.1371/journal.pone.0099430)

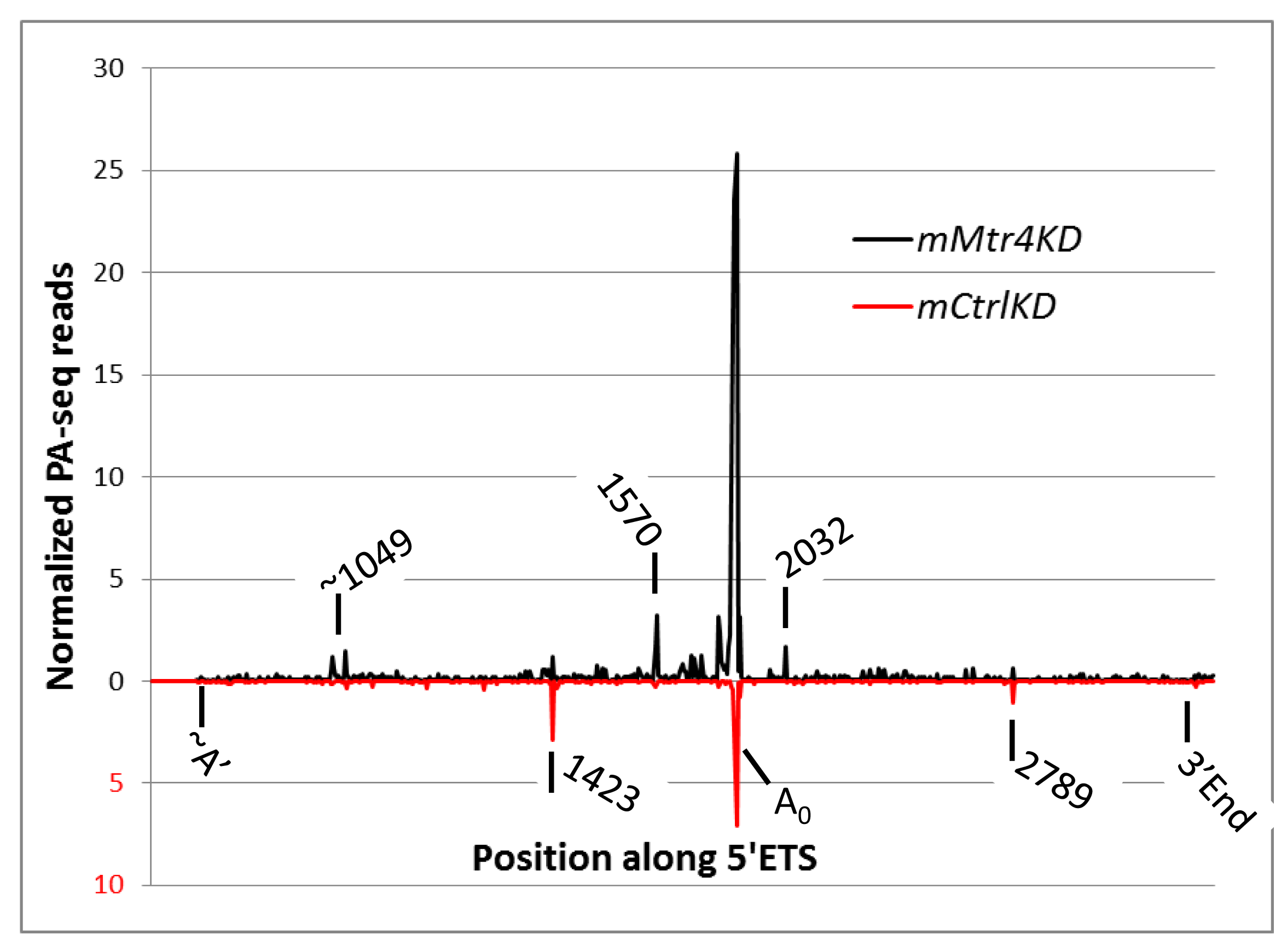

Supplement: Figure S1 — Density of PA-seq reads across the entire 5′ ETS. Graph depicts the level of adenylation at individual positions across the 4 kb 5′ ETS. Positions for which zero reads were detected in either the mMtr4KD or mControlKD were omitted for improved resolution of all remaining positions. Positions along the x-axis are not to scale, but several reference points are indicated. (TIF) [file pone.0099430.s001.tif]

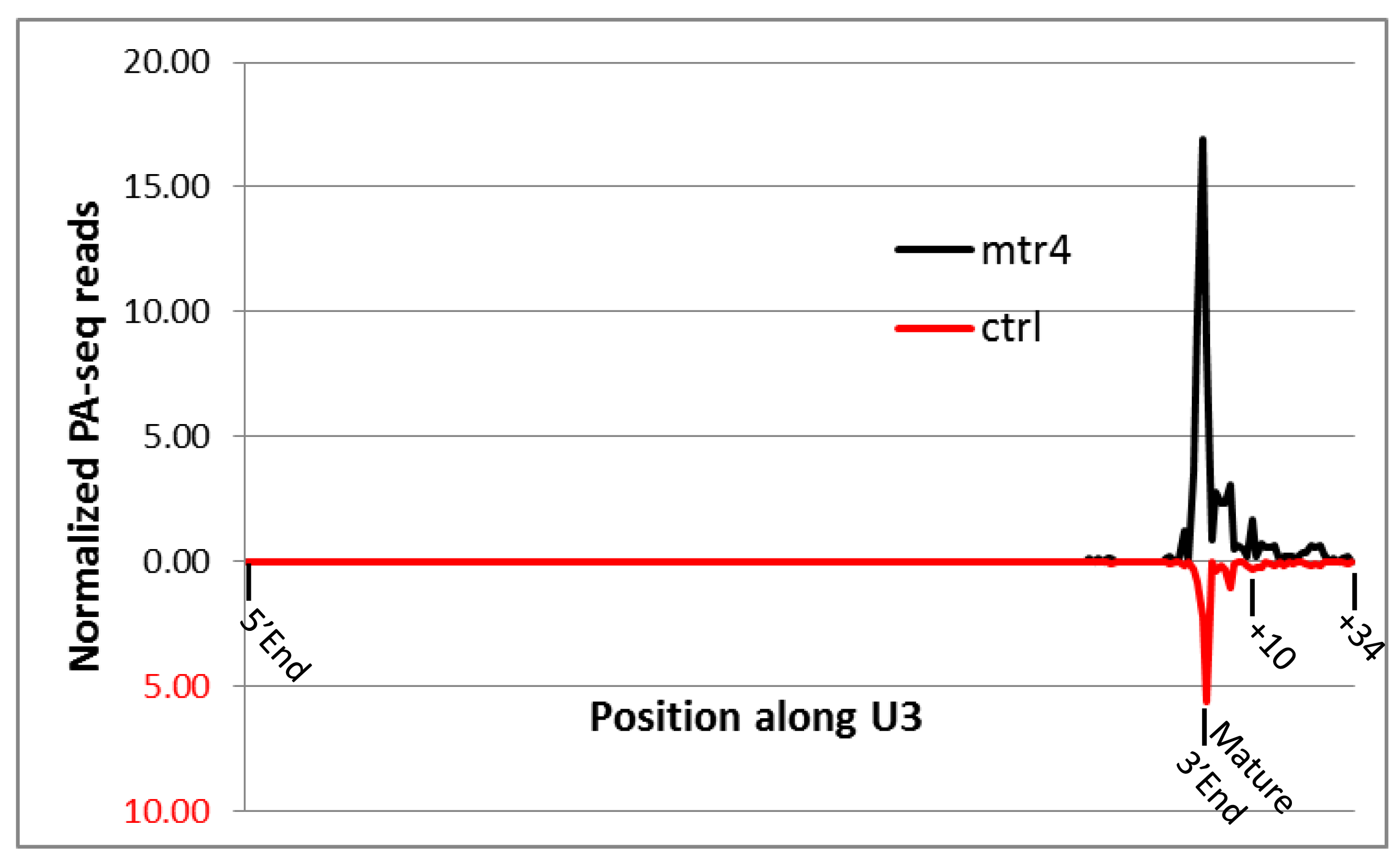

Supplement: Figure S2 — Density of PA-seq reads encompassing U3B. Graph depicts the level of adenylation at individual positions across the full length of U3B snoRNA. Data are pooled for the four tandem copies of U3B found on chromosome 11 of the mouse genome. (TIF) [file pone.0099430.s002.tif]
